# Difference in Aneurysm Characteristics between Patients with Familial and Sporadic Aneurysmal Subarachnoid Haemorrhage

**DOI:** 10.1371/journal.pone.0154281

**Published:** 2016-04-22

**Authors:** Liselore A. Mensing, Gabriel J. E. Rinkel, Monique H. M. Vlak, Irene C. van der Schaaf, Ynte M. Ruigrok

**Affiliations:** 1 Department of Neurology and Neurosurgery, Brain Center Rudolf Magnus, University Medical Center Utrecht, Utrecht, the Netherlands; 2 Department of Radiology, University Medical Center Utrecht, Utrecht, the Netherlands; 3 Department of Neurology, Medical Center Haaglanden, The Hague, the Netherlands; Stellenbosch University Faculty of Medicine and Health Sciences, SOUTH AFRICA

## Abstract

**Object:**

Patients with familial intracranial aneurysms (IA) have a higher risk of rupture than patients with sporadic IA. We compared geometric and morphological risk factors for aneurysmal rupture between patients with familial and sporadic aneurysmal subarachnoid hemorrhage (aSAH) to analyse if these risk factors contribute to the increased rupture rate of familial IA.

**Methods:**

Geometric and morphological aneurysm characteristics were studied on CT-angiography in a prospectively collected series of patients with familial and sporadic aSAH, admitted between September 2006 and September 2009, and additional patients with familial aSAH retrieved from the prospectively collected database of familial IA patients of our center. Odds ratios (OR) with corresponding 95% confidence intervals (95% CI) were calculated to compare the aneurysm characteristics between patients with familial and sporadic aSAH.

**Results:**

We studied 67 patients with familial and 184 with sporadic aSAH. OR’s for familial compared with sporadic aSAH were for oval shape 1.16(95%CI:0.65–2.09), oblong shape 0.26(95%CI:0.03–2.13), irregular shape 0.83(95%CI:0.47–1.49), aspect ratio ≥ 1.6 0.94(95%CI:0.54–1.66), contact with the perianeurysmal environment (PAE) 1.15(95%CI:0.56–2.40), deformation by the PAE 1.05(95%CI:0.47–2.35) and for dominance of the posterior communicating artery (PCoA) in case of PCoA aneurysms 1.97(95% CI:0.50–7.83).

**Conclusions:**

The geometric and morphological risk factors for aneurysm rupture do not have a higher prevalence in familial than in sporadic aSAH and thus do not explain the increased risk of IA rupture in patients with familial IA. We recommend further search for other potential risk factors for rupture of familial IA, such as genetic factors.

## Introduction

Familial predisposition is the strongest risk factor for aneurysmal subarachnoid hemorrhage (aSAH).[1] A report from the Familial Intracranial Aneurysm (FIA) study found a 17-times higher rupture rate for patients with familial intracranial aneurysms (IA) compared to patients with sporadic IA matched for age, gender, location and size of the aneurysms.[2] The cause of this increased rupture rate of familial IA is as yet unknown.

Recently, a meta-analysis of six prospective cohort studies on risk of rupture showed that prognostic factors for IA rupture include age, hypertension, history of aSAH, geographical region and IA size and location, with IA > 7 mm and IA in the vertebrobasilar, anterior communicating and posterior communicating arteries carrying the highest risk of rupture.[3] Previous studies suggest that patients with familial IA are younger and have larger IA at time of rupture and more often have multiple IA and IA located at the middle cerebral artery.[4–7] The presence of hypertension does not differ between patients with familial and sporadic IA,[8] while no data on a possible difference in previous history of aSAH exist. Therefore, of the afore mentioned prognostic factors, only IA size may contribute to the higher risk of rupture of familial IA and a further search for risk factors contributing to the increased rupture rate is warranted.

Suggested additional geometric and morphological risk factors for IA rupture include aneurysmal shape, various size and shape ratio’s, contact between the aneurysmal wall and surrounding anatomic structures and dominance of the posterior communicating artery (PCoA) in case of PCoA IA.[3,9,10]

In this study we compared geometric and morphological risk factors for aneurysmal rupture between patients with familial aSAH and patients with sporadic aSAH to analyse if these risk factors contribute to the increased rupture rate of familial IA.

## Methods

### Study population

From a prospectively collected cohort of 250 consecutive aSAH patients admitted to the University Medical Center Utrecht (UMCU) between September 2006 and September 2009, we compared patients with familial aSAH to patients with sporadic aSAH.[11] In addition, we used the cohort of familial aSAH patients admitted between January 2003 and September 2006 and between October 2009 and January 2014, retrieved from the prospectively collected database of familial IA patients of the UMCU. The Medical Ethical Committee of the University Medical Center Utrecht approved the data collection used, and written informed consent was obtained. Familial aSAH was defined as two or more first degree relatives with definite or probable aSAH. Definite aSAH was defined as an abrupt onset of severe headache or loss of consciousness with or without focal neurological signs, the presence of subarachnoid blood on head CT compatible with a ruptured aneurysm and an aneurysm on CT-angiography (CTA), magnetic resonance angiography (MRA) or digital subtraction angiography (DSA). Probable aSAH was defined as either sudden severe headache in combination with a normal neurological examination and hemorrhagic CSF, followed by sudden deterioration and death within 4 weeks (consistent with rebleeding), or as a history describing a second ictus followed by death within the first 4 weeks after “stroke” and age < 70 years.[12] Exclusion criteria were: 1) unavailable or poor quality CTA; 2) fusiform IA; 3) inability to identify the location of the ruptured IA in case of multiple IA; 4) previous history of conditions known to predispose to IA formation.[13]

### Data extraction and imaging

The geometric and morphological aneurysm characteristics were reviewed on CTA images of the circle of Willis. The CTA scans were performed with a field of view of 160 mm and a slice thickness of 1.0 mm reconstructed at 0.5 mm. CTA source image data of all patients were transferred to an offline workstation (IntelliSpace Portal, v6.0.1.20250, Philips Healthcare) for interactive viewing and post-processing. CTA scans were reviewed blinded for family history by the same observer (LAM). Complex cases were discussed in a consensus meeting with an experienced neuroradiologist (ICvdS). A standardized window setting (window level and window width equal to the Hounsfield units within the aneurysm) was used to perform all measurements. The images could be rotated in three dimensions for all measurements and volume rendering was used for evaluation of the perianeurysmal environment (PAE).

### Definitions of variables

#### Aneurysmal shape

Shape of the IA was divided into spherical (width > 80% of length) or elliptical (width < 80% of length), which was further divided into oval (width 50–80% of length) and oblong (width < 50% of length).[14] IA were considered to have an irregular shape when multiple lobes, a bleb or daughter sac were present.

#### Aspect ratio

Aspect ratio is used to describe the relation between the length and the neck of the IA and is calculated by dividing the maximal neck-to-dome-length by the neck-width using a 0.1-point scale. Aspect ratio was dichotomized into < 1.6 and ≥ 1.6.[15–17]

#### Perianeurysmal environment

**Fig 1:**
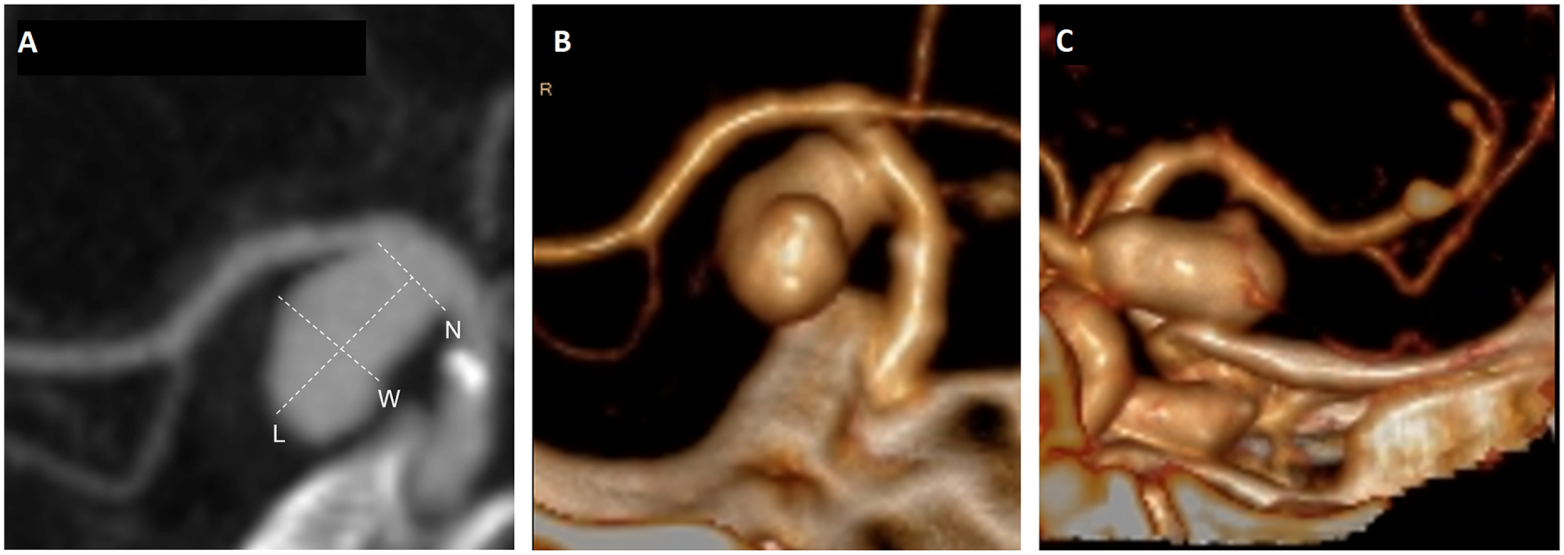
Definitions of aneurysm-related risk factors. **Panel A** Aneurysmal size of a right internal carotid artery aneurysm: N = neck (maximal length of the segment adjacent to the orifice), L = length (distance between neck center and dome of the aneurysm), W = width (largest distance perpendicular to length); **Panel B and C** Contact of a right internal carotid artery aneurysm with the perianeurysmal environment: coronal **(B)** and sagittal view **(C)** showing flattening of a right internal carotid artery aneurysm draping over the bony sella turcica.

The aneurysm wall was evaluated for contact with bone or vessels in the PAE using volume rendering (Fig 1). Deformation of the aneurysm by the PAE was defined as a local change in contour of the aneurysm wall at the location of contact with a structure in the PAE or as a protrusion of the aneurysm wall contralateral of the location of contact with a structure in the PAE.[9] Three categories of PAE interaction were defined: 1) no contact with the PAE, 2) contact with the PAE without deformation of the IA and 3) deformation of the IA by contact with the PAE.

#### Dominance of the PCoA in case of PCoA aneurysms

For patients with familial or sporadic PCoA IA, dominance of the PCoA was studied on CTA. Vessel diameter of the PCoA and P1-segment of the posterior cerebral artery (PCA) were measured ipsilateral of the IA. The PCoA was considered dominant if the PCoA diameter exceeded the diameter of the P1-segment of the PCA with more than 33%.

### Data analysis

We calculated odds ratio’s (ORs) with corresponding 95% confidence intervals (CI) to compare aneurysmal shape, aspect ratio ≥ 1.6, contact with or deformation of the IA by the PAE, and dominance of the PCoA in case of PCoA IA between patients with familial and sporadic aSAH. Multivariable logistic regression analysis was used to adjust for possible confounding by the six factors known to be associated with IA rupture: age, gender, previous aSAH, hypertension and IA size and location.[3] We did not adjust for IA location in the analysis of dominance of the PCoA. First, analyses were performed comparing all included patients with familial aSAH with patients with sporadic aSAH. Second, to test for possible selection bias, sensitivity analyses were performed using only the prospectively collected cohort of consecutive familial and sporadic aSAH patients [11] and thus excluding the additional cohort of familial aSAH patients from the prospectively collected database of familial IA patients.

## Results

In total, 22 patients with familial and 13 patients with sporadic aSAH were excluded for the following reasons: good quality CTA was not available for analysis (n = 23), CTA showed a fusiform IA (n = 6), the ruptured IA could not be identified in case of multiple IA (n = 5) or the patient had a history of polycystic kidney disease (n = 1). Baseline characteristics of the remaining 67 patients with familial aSAH and 184 patients with sporadic aSAH included in the analysis are summarized in Table 1.

**Table 1:** Baseline characteristics of the 67 patients with familial and 184 patients with sporadic aneurysmal subarachnoid hemorrhage.

Characteristics	Familial aSAH (n = 67) n (%)	Sporadic aSAH (n = 184) n (%)
Women	54 (81)	135 (73)
Mean age*, y (SD)	55 (12)	54 (12)
Hypertension	18 (27)	41 (22)
Smoking*, (n = 61/184)	39 (64)	111 (60)
Aneurysm size		
≥ 7 mm	27 (40)	98 (53)
Aneurysm location		
ACA/ACoA/PeriA	20 (30)	79 (43)
ICA	9 (13)	10 (5)
PCoA	11 (16)	37 (20)
MCA	16 (24)	38 (21)
BA/VA	11 (16)	20 (11)

*ACA* anterior cerebral artery, *ACoA* anterior communicating artery, *BA* basilar artery, *ICA* internal carotid artery, *MCA* middle cerebral artery, *n* number, *PCoA* posterior communicating artery, *PeriA* pericallosal artery, *SD* standard deviation, *VA* vertebral artery, *y* years,

* at time of aSAH

Of the 67 patients with familial aSAH, 38 were identified from the prospectively collected cohort of consecutive aSAH patients [11] and 29 from the prospectively collected database of familial IA patients.

Aneurysmal shape, aspect ratio ≥ 1.6, contact with or deformation of the IA by the PAE, and dominance of the PCoA in case of PCoA IA were not significantly associated with familial aSAH (Table 2).

**Table 2:** Geometric and morphological aneurysm characteristics in the 67 patients with familial and 184 patients with sporadic aneurysmal subarachnoid hemorrhage.

Characteristics	Familial aSAH (n = 67)	Sporadic aSAH (n = 184)		
	n (%)	n (%)	OR (95% CI)	aOR (95% CI)
*Shape*				
Spherical	24 (36)	69 (38)	Reference	Reference
Elliptical				
–oval	42 (63)	104 (57)	1.16 (0.65–2.09)	1.29 (0.69–2.41)
–oblong	1 (2)	11 (6)	0.26 (0.03–2.13)	0.35 (0.04–3.20)
*Shape*				
Irregular shape	42 (63)	123 (67)	0.83 (0.47–1.49)	1.21 (0.63–2.35)
*Aspect ratio*				
≥ 1.6	38 (57)	107 (58)	0.94 (0.54–1.66)	1.36 (0.71–2.62)
*Perianeurysmal environment*				
No contact or deformation	44 (66)	125 (68)	Reference	Reference
Contact (without deformation)	13 (19)	32 (17)	1.15 (0.56–2.40)	1.27 (0.58–2.73)
Contact and deformation	10 (19)	27 (15)	1.05 (0.47–2.35)	1.28 (0.53–3.12)
*PCoA aneurysms (n = 48)*				
PCoA dominance	5 (46)	11 (30)	1.97 (0.50–7.83)	0.40 (0.09–1.88)

*(a)OR* (adjusted) Odds Ratio, *aSAH* aneurysmal subarachnoid hemorrhage, *CI* confidence interval, *PCoA* posterior communicating artery

These results did not change after adjustment for age, gender, previous aSAH, hypertension, IA size and location. When comparing only patients with familial and sporadic aSAH from the prospectively collected cohort of consecutive aSAH patients [11] the results were essentially the same (data not shown).

## Discussion

Our study shows that geometric and morphological aneurysm characteristics associated with a higher rupture rate of IA, e.g. aneurysmal shape, aspect ratio ≥ 1.6, contact with or deformation by the PAE, and dominance of the PCoA in case of PCoA IA do not differ between patients with familial aSAH as compared with patients with sporadic aSAH. Therefore, these characteristics do not explain the increased risk of IA rupture in patients with familial IA as compared to patients with sporadic IA.

First degree relatives of patients with familial aSAH are advised to be screened for unruptured IA. In case an unruptured IA is discovered, knowledge on risk factors for rupture of familial IA is essential to select those relatives at high risk of IA rupture who could benefit from preventive treatment. Our results imply that the geometric and morphological aneurysm characteristics studied will not contribute in detecting these high-risk first degree relatives of patients with familial IA. Thus far, only IA size has been found as an explanatory factor for the higher risk of rupture of familial IA,[6] although not all studies found a larger aneurysm size at rupture in familial than in sporadic IA.[4,5] Other potential risk factors include genetic factors. To date no genetic factors associated with IA rupture have been found, since most genetic studies performed thus far have not made a distinction between patients with unruptured and ruptured IA. Future studies should focus on the identification of genetic factors associated with rupture, their potential difference between patients with sporadic and familial IA, and the existence of gene-environment interactions [18] to clarify the increased risk of rupture of familial IA.

A strength of the current study is that all characteristics were studied on CTA images using the same structured approach. Furthermore, data collection and review of CTA scans was performed blinded for family history to prevent observer bias. Our study also has limitations that need to be addressed. First, we did not find a difference in prevalence of aneurysm characteristics associated with rupture studying a relatively small number of patients. Therefore, the results of this study should be considered preliminary. However, considering this number of included patients we were able to exclude a mean difference in the prevalence of aneurysm characteristics associated with rupture larger than 20% between sporadic and familial aSAH assuming a beta of 0.80. Second, patients and controls were not matched for IA size and location, which are important risk factors for rupture. Therefore we adjusted for these characteristics in a multivariate analysis. Third, there are several new techniques to study aneurysm shape, such as the ellipticity index, nonsphericity index and the undulation index, but for this study we have chosen the method that is most widely used at this time. Fourth, we restricted evaluation of the PAE to visible structures on CTA scans such as bone or vessels. This might have led to an underestimation of the actual interaction, as we might have missed other structures in the PAE modulating the shape of the IA and thereby causing the IA to rupture. But we do not expect to have missed a difference in interaction with the PAE between patients with familial and sporadic IA, since CTA scans were assessed in the same structured manner for both groups.

## Conclusions

The geometric and morphological risk factors for aneurysm rupture do not have a higher prevalence in familial than in sporadic aSAH and thus do not explain the increased risk of IA rupture in patients with familial IA. We recommend further search for other potential risk factors for rupture of familial IA, such as genetic factors. Knowledge on these risk factors will help to identify those first degree relatives of patients with familial IA at high risk of rupture of an IA, for whom preventive treatment should be considered.

## Supporting Information

S1 File Dataset containing baseline and aneurysm characteristics of patients with familial and sporadic aneurysmal subarachnoid haemorrhage. (XLSX)
